# Microfluidics-on-a-chip for designing celecoxib-based amorphous solid dispersions: when the process shapes the product

**DOI:** 10.1007/s13346-024-01633-7

**Published:** 2024-06-11

**Authors:** Joana Figueiredo, Maria Mendes, Alberto Pais, João Sousa, Carla Vitorino

**Affiliations:** 1 Faculty of Pharmacy, University of Coimbra, Pólo das Ciências da Saúde, Azinhaga de Santa Comba, 3000-548 Coimbra, Portugal; 2 Coimbra Chemistry Centre, Institute of Molecular Sciences - IMS, Department of Chemistry, University of Coimbra, 3004-535 Coimbra, Portugal

**Keywords:** Amorphous solid dispersions, Microfluidics-on-chip, Bioavailability, Poorly water-soluble drugs, Polymers, Performance

## Abstract

The fundamental idea underlying the use of amorphous solid dispersions (ASDs) is to make the most of the solubility advantage of the amorphous form of a drug. However, the drug stability becomes compromised due to the higher free energy and disorder of molecular packing in the amorphous phase, leading to crystallization. Polymers are used as a matrix to form a stable homogeneous amorphous system to overcome the stability concern. The present work aims to design ASD-based formulations under the umbrella of quality by design principles for improving oral drug bioavailability, using celecoxib (CXB) as a model drug. ASDs were prepared from selected polymers and tested both individually and in combinations, using various manufacturing techniques: high-shear homogenization, high-pressure homogenization, microfluidics-on-a-chip, and spray drying. The resulting dispersions were further optimized, resorting to a 3^2^ full-factorial design, considering the drug:polymers ratio and the total solid content as variables. The formulated products were evaluated regarding analytical centrifugation and the influence of the different polymers on the intrinsic dissolution rate of the CXB-ASDs. Microfluidics-on-a-chip led to the amorphous status of the formulation. The in vitro evaluation demonstrated a remarkable 26-fold enhancement in the intrinsic dissolution rate, and the translation of this formulation into tablets as the final dosage form is consistent with the observed performance enhancement. These findings are supported by ex vivo assays, which exhibited a two-fold increase in permeability compared to pure CXB. This study tackles the bioavailability hurdles encountered with diverse active compounds, offering insights into the development of more effective drug delivery platforms.

**Graphical Abstract:**

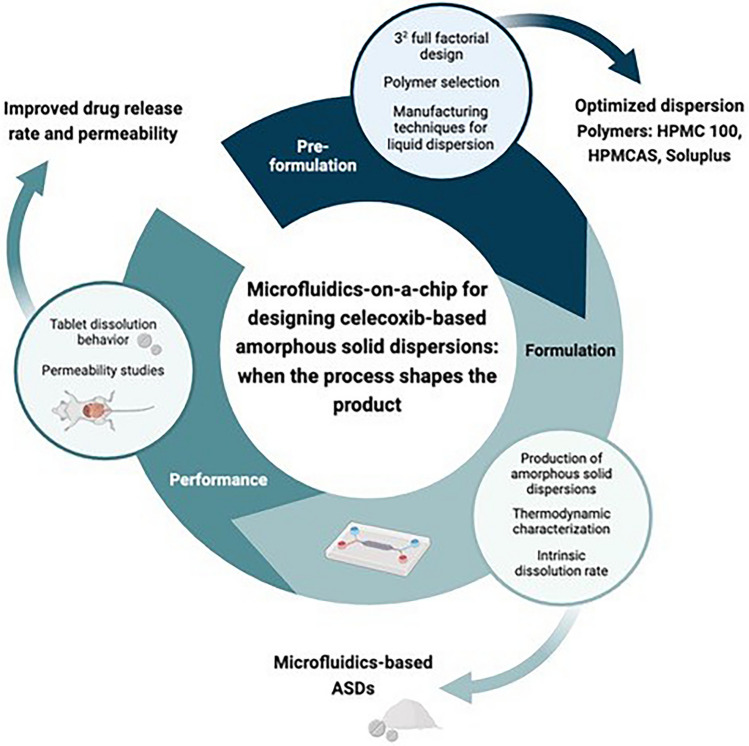

## Introduction

More than half of the pharmaceutical global market share is represented by oral formulations. The oral route is the most common drug administration route due to its advantages [1]. Among them are the simplicity of drug administration, patient compliance, cost-effectiveness, and the ease of manufacturing scale-up. Moreover, advances in pharmaceutical technology have enabled the precise targeting of drugs to specific sites of interest. This means that orally administered active pharmaceutical ingredients (APIs) can selectively target particular regions of the gastrointestinal tract (GIT) [2]. Notwithstanding the apparent advantages, the development of oral formulations remains challenging due to the GIT microenvironment and physiological barriers, including pH, metabolic enzymes, and efflux transporters [3, 4]. Additionally, the physicochemical properties of drugs also play a role in the hurdles of oral drug delivery, considering that many drug candidates exhibit suboptimal biopharmaceutical characteristics for oral dosing. The Biopharmaceutics Classification System (BCS) is a valuable scientific approach that involves mathematical analysis to determine the aqueous solubility and intestinal permeability characteristics of active substances, categorizing drug substances in four classes [5]. For instance, a significant number of new chemical entities are listed in the BCS class II, III, or IV, wherein solubility and/or intestinal permeation can limit absorption [6].

During the last decades, both academic and industrial research has been demonstrating the potential of amorphous solid dispersions (ASDs) in overcoming the challenge posed by poor water solubility of drugs. The amorphous form exhibits a disarrangement in the crystal lattice of its crystalline counterpart, generating a higher state of free energy, allowing the drug to achieve greater apparent solubility and faster dissolution [7]. However, the amorphous form is metastable and tends to convert to a more stable crystalline form. Thus, polymers are usually required to delay or inhibit recrystallization and ensure the benefits of this high-energy state. The addition of polymers to a solid dispersion can reduce the molecular mobility of pharmaceuticals, thereby inhibiting crystallization. This process also induces an antiplasticizing effect and elevates the glass transition temperature of the system [8]. Polyvinylpyrrolidone (PVP), polyvinylpyrrolidone-co-vinyl acetate (PVPVA), hydroxypropyl methylcellulose (HPMC), and hydroxypropyl methylcellulose acetate succinate (HPMCAS) are among the most often used polymers. Distinct polymers can assign different benefits and drawbacks in the development of ASDs [9]. One approach to optimize the desired characteristics of these systems is by combining different polymers, thereby utilizing polymer blends as excipients [10].

Celecoxib (CXB) is a non-steroidal anti-inflammatory drug that selectively inhibits cyclooxygenase-2 (COX-2). It falls under the Biopharmaceutical Classification System (BCS) class-II category due to its low water solubility and high membrane permeability, and it exhibits three polymorphic forms, consequently compromising its bioavailability [11]. Celecoxib (CXB) was selected as a model drug due to its poorly water-soluble properties, making it an ideal candidate for studying amorphous solid dispersion (ASD) formulations aimed at enhancing both physical stability and aqueous solubility. Despite being weakly basic and soluble in gastric medium in its crystalline state, CXB provides an excellent model compound for studying the mechanisms involved in improving its solubility profile through ASDs. CXB is particularly suited because it exhibits low solubility (1.5 µg/mL) in aqueous solutions at pH 6.8 and high hydrophobicity (logP = 3.4), placing it within BCS class II [12–14]. Additionally, it represents a BCS class II drug due to its low aqueous solubility (5 µg/mL at 5–40 °C) and high permeability properties [15]. Notably, its solubility remains independent of pH below 9.0 but increases significantly at pH 12 [16]. Therefore, comparing the amorphous form of CXB with its crystalline form presents an excellent opportunity to explore its solubility enhancement potential further.

The aim of this study was to address the challenges associated with CXB by proposing formulations using it as ASDs through various technological approaches. These strategies were guided by quality by design principles to enhance drug bioavailability. Different preparation methods were considered, including high-shear homogenization, high-pressure homogenization, spray drying, and flow-focused microfluidics-on-a-chip as a differentiated production method. Microfluidics-on-a-chip relies on the nanoprecipitation process, enabling precise control over the mixing rate between solvent and antisolvent phases. This confers an advantage over conventional precipitation methods by facilitating tunability of mixing dynamics. Consequently, the production of amorphous drug nanoparticle yields a more uniformly dispersed system with reduced particle sizes, thereby increasing the surface area of the particles. Material sparing and small sample size are other factors in favor of microfluidics, as miniaturization of the process allows for a reduction in the consumption of formulation components in the screening stage [17]. After the formulation process, it was essential to characterize the solid-state of the obtained ASDs to move on to downstream processing. Solid-state characterization techniques such as differential scanning calorimetry (DSC), X-ray powder diffraction (XRPD), Fourier transform infrared spectroscopy (FTIR), and scanning electron microscopy (SEM) were commonly employed to assess possible recrystallization within ASDs, as well as to investigate miscibility, chemical interactions between API and polymers, and particle morphology. Finally, a comprehensive understanding of drug release was obtained through dissolution tests and ex vivo studies to elucidate the advantages of using ASDs and evaluate their performance, stability, and potential for enhancing drug bioavailability.

This comparative research between microfluidics-on-a-chip and conventional production methods underlines the potential of microfluidics as a promising alternative for the production of ASDs.

## Materials and methods

### Materials

Celecoxib (98.0 ~ 102.0% purity) was purchased from Shandong Zhishang Chem Co., Ltd (Shandong Province, China). Hydroxypropyl methylcellulose (HPMC) of grades 4, 100, 4000, 15000, and 100000 were donated by Shin-Etsu (Shin-Etsu, Japan), while hydroxypropyl methylcellulose acetate succinate (HPMCAS) was kindly offered by Seppic (Seppic SA, France). Soluplus ^®^ was acquired from BASF (Rheinland-Pfalz, Germany), polyvinyl pyrrolidone K30 (PVP K30) was purchased from Fluka (Missouri, USA), polyvinyl alcohol (PVA) and polyethylene glycol 400 (PEG 400) were purchased from Sigma-Aldrich (Missouri, USA). Water was ultrapurified using a Milli-Q water apparatus (Millipore^®^, USA) and filtered through a 0.22 μm nylon filter before use. All other reagents and solvents were from analytical or high-performance liquid chromatography grade.

### Methods

#### Quality target product profile and initial risk assessment

The Quality Target Product Profile (QTPP) is a fundamental element of the QbD approach, wherein the essential attributes a drug product should attain are prospectively summarized, envisioning the quality features intended to be reached, with a focus on ensuring the safety and efficacy of the drug product [18, 19]. A risk estimation matrix (REM) was constructed to identify and prioritize potential high-risk process parameters that could influence the critical quality attributes.

#### High-throughput screening of polymers

The study aimed to streamline high throughput screening assays involving five distinct polymers, including various grades and celecoxib (CXB), at a concentration of 1% (w/V) in sample volumes of 10 mL. The drug and polymers were dissolved in different solvents. The preferred solvent was ethanol due to its ability to solubilize CXB and the polymers. A mixture of ethanol and dichloromethane (2:1) was used when the polymers were not soluble in ethanol, which was the case for all HPMC and PVA molecular weights. The solutions were dispersed into the 24-well plates (n = 3), evaporating the solvent. Subsequent to solvent evaporation, the solutions were incubated at 37 °C for 2h with a dissolution media composed of 1.5 mL of HCl 0.01 N + PEG 400 (70:30, % V/V) to represent the gastric fluid pH in the fasted state. The solubilization capacity of the carriers for CXB was determined using a high-performance liquid chromatography (HPLC) method previously validated according to the FDA and ICH recommendations [20].

#### 3^k^ full-factorial design

A three-level full factorial design with two variables, 3^2^, was used to study the effect of formulation variables on critical quality attributes (CQAs) of solid dispersions. Two factors were considered, each at low, intermediate, and high levels, numerically expressed as -1, 0, and +1, respectively. A central point is included to understand the model curvature in the response function and investigate a quadratic relationship between the response and each factor [21]. The optimal conditions were selected based on the quadratic polynomial function represented in

1 $$\begin{aligned}Y\,&=\,{\beta }_{0}\,+\, {\beta }_{1}{x}_{1}\,+\, {\beta }_{2}{x}_{2}\,+\, {\beta }_{12}{x}_{1}{x}_{2}\,\\&+\, {{\beta }_{11}{x}_{11}}^{2}\,+\, {{\beta }_{22}{x}_{22}}^{2}\end{aligned}$$

where Y is the measured response, ${\beta }_{0}$ is the response in the absence of effects, ${\beta }_{1}$ and ${\beta }_{2}$ are the coefficients of the respective independent variables, ${\beta }_{12}$ is the interaction coefficient between the two factors, and ${\beta }_{11}$ and ${\beta }_{22}$ are the quadratic coefficients obtained from the observed experimental values of Y, ${x}_{1}$ and ${x}_{2}$ are the terms representing the two factors and ${x}_{11}$ and ${x}_{22}$ represent the quadratic terms [20, 22].

The two independent variables, displayed in Table 1, were the total solid content and the ratio of Drug:Polymer 1:Polymer 2. As responses, particle size, polydispersity index, and assay were considered. The experimental design was solved using the JMP Statistical Analysis Software, JMP Pro version 17.0.0. Both the Student t-test and ANOVA were conducted to assess the significance of the regression model terms and evaluate the model fit.

**Table 1:** Experimental design independent variables and respective codification

**Formulation Parameters**	**Codification**	**Levels**		
		**-1**	**0**	** +1**
**Total solid content**	${\beta }_{1}$	0.75%	1.5%	2.25%
**Drug:P1:P2 ratio**	${\beta }_{2}$	1:1:1	1.5:0.75:0.75	2:0.5:0.5

*P1* Polymer 1, *P2* Polymer 2

#### Analytical centrifugation

The LUMiSizer equipment (LUM GmbH, Berlin, Germany) allowed a predictive assessment of the formulations’ physical stability behavior. This multisample analytical centrifuge enables the measurement of the intensity of transmitted near-infrared light as a function of time and position through the entire sample extent. The transmission profiles provide information relative to the kinetics of separation processes, allowing sedimentation and/or creaming rates to be determined. The instability index, a quantitative parameter, also acknowledges the evaluation of formulation stability and ranges from 0 to 1. A value of 0 suggests formulation stability with no changes under test conditions, whereas a value of 1 indicates greater instability of the formulations, revealing different instability phenomena represented in the transmission profiles [23, 24]. The stability of the formulations was analyzed after 1h30 of centrifugation at an acceleration of 4000 rpm and 25 °C. The determination of stability parameters was performed using the SEPView^®^ software.

#### Preparation of dispersions by antisolvent co-precipitation

As represented in Table 2, three different formulations were prepared using the antisolvent precipitation technique. When required, according to the formulation composition, the solvent (S) organic phase containing CXB and HPMCAS-HG was prepared by dissolving the components in ethanol due to their insolubility in water. The remaining polymers were dissolved in ultrapurified water, constituting the antisolvent (AS) aqueous phase.

**Table 2:** Pre-formulation compositions

**F**	**Drug** **(0.75% w/V)**	**Polymer 1** **(0.75% w/V)**	**Polymer 2** **(0.75% w/V)**
**1**	Celecoxib	PVP K30	Soluplus^®^
**2**		HPMC 100	Soluplus^®^
**3**		HPMCAS-HG	Soluplus^®^

##### High-shear homogenization

Dispersions by antisolvent precipitation were obtained by introducing the antisolvent aqueous phase in the solvent organic phase directly under a high-speed stirrer (Ultra-Turrax X1020; Ystral GmbH, Dottingen, Germany) at 11000 rpm for 15 min. Subsequently, the suspension formulation was diluted with ultrapurified water to analyze particle size and polydispersity index.

##### High-pressure homogenization

After being submitted to high-shear homogenization, the dispersions were further processed in a high-pressure homogenizer (HPH) (Emulsiflex^®^-C3, Avestin, Inc., Ottawa, Canada) at 25ºC and a pressure of 1000 bar for 15 min. The obtained formulation was subsequently diluted with ultrapurified water to analyze particle size and polydispersity index.

##### Spray drying

Amorphous solid dispersions obtained by spray drying were manufactured using a Mini Spray Drier B-290 (BÜCHI Labortechnik AG), equipped with a two-fluid nozzle (∅ 0.7 mm, stainless steel). Temperature conditions of the spray drier were an inlet temperature of 140 °C and an outlet temperature of 45 ± 5 °C [25]. The peristaltic pump was set at 30% (approximately 9 mL/min), and the aspirator rate was set at 100% (approximately 35 m^3^/h). Automatic nozzle cleaning was used throughout the whole experiment.

##### Microfluidics-on-a-chip

Microfluidics-on-a-chip was here considered as an alternative technique. Briefly, the organic and aqueous phases described in Table 2 were respectively pumped into two reservoirs connected to the inlet channels of a pressure-driven flow control system Elveflow OB1 MK3+ (Elvesys, Paris, France) at various flow rates to obtain S:AS ratios of 1:1, 1:2, 1:4, 1:8 and 1:10. The rapid mixing of solvent and antisolvent streams occurred in a Y-junction herringbone microfluidics microchip (200 μm depth, 188 μm thickness). The obtained dispersions were subsequently diluted with ultrapurified water to analyze particle size and polydispersity index. Before and after each formulation development, pure solvents were run through the equipment to clean the system and prevent contamination thoroughly. The pressure was systematically monitored to guarantee the same conditions for every formulation.

#### Analytical methods for characterization of amorphous solid dispersions

Characterizing the properties of ASDs is crucial in all stages of product development. This allows us to understand molecular interactions and rationally select the formulation composition and processing methods, obtaining high-quality products [26].

##### Particle size analysis

Evaluation of particle size is of utmost importance since it has been demonstrated that it is inversely proportional to the dissolution rate [27]. Dynamic light scattering and laser diffraction were considered depending on the sample size range.

##### Dynamic light scattering

Dynamic light scattering (DLS) is a well-established technique for determining the size of particles in the submicron (< 1µm) region. Alongside particle size, the polydispersity index is a parameter that should be considered when defining the particle size distribution. The formulations’ particle size was determined using a Zetasizer Nano ZS, Malvern (Malvern, Worcestershire, UK) set at a detection angle of 173° and a temperature of 25 °C. All samples were diluted 100 times in ultrapurified water and analyzed in triplicate.

##### Laser diffraction

The laser diffraction measures volume-weighted particle size distributions, and the parameters are reported based upon percentiles, of which D_v__10_, D_v__50_, and D_v__90_ are the most commonly portrayed. The span value is an additional parameter that indicates the size distribution width. It is calculated according to

2 $$\text{Span }\,=\, \frac{\text{D}_{v90} \,-\,\text{ D}_{v10}}{\text{D}_{v50}}$$

Laser diffraction was performed using the MasterSizer 3000 equipped with the Aero S accessory (Malvern, Worcestershire, UK). The sample was placed inside the Aero S, and the equipment performed background evaluation and system alignment. An obscuration range of 0.1 – 10% was used. The real and imaginary refractive indices were set to 1.61 and 0.01, respectively. Six measurements were carried out for each sample.

##### Differential scanning calorimetry

Differential scanning calorimetry (DSC) was performed in a DSC-204 F1 Phoenix differential scanning calorimeter (Netzsch, Germany) to assess the thermal properties of pure compounds and amorphous solid dispersions. Samples of 2 mg were weighted and placed in aluminum pans that were hermetically sealed. Each sample was heated at a heating rate of 10 °C/min from 25 to 200 °C, with a nitrogen purge of 20 mL/min (Table 3).

**Table 3:** Melting temperature of active pharmaceutical ingredient and glass transition temperatures of the polymers

**Drug/Polymer**	**T**_**melting**_** (°C)**	**T**_**glass-transition**_** (°C)**
**CXB**	161 – 164	
**HPMC 100**		175 – 185
**HPMCAS**		135
**Soluplus**^**®**^		70
**PVP K30**		163

##### Attenuated total reflectance Fourier-transform infrared

Attenuated total reflectance infrared spectroscopy (ATR-FTIR) spectra were obtained using a FT-IR/FT-NIR spectrometer (Spectrum 400, PerkinElmer, MA, USA) equipped with a crystal diamond ATR sampling accessory. Pure compounds and samples were placed on top of the ATR device and measured using 32 scans for each spectrum, recorded in the 4000–650 cm^-1^ range with a 2 cm^-1^ resolution.

##### X-ray powder diffraction

X-ray powder diffraction (XRPD) diffractograms were obtained using a Rigaku MiniFlex 600 diffractometer (Rigaku, Japan) to assess the amorphization state of the samples and crystallinity of pure compounds. Samples were placed in zero background holders, and spin was used to avoid preferential orientation. In all measurements, Cu K_α_ radiation (λ = 1.541862 Å) was used.

##### Scanning electron microscopy

Optimized formulations were analyzed by scanning electron microscopy (SEM). A JSM 6010LV/6010LA, Jeol (Tokyo, Japan) was used to determine the morphology of the samples. Prior to analysis, the powder was spread on a double-sided carbon tape mounted onto an aluminum stud. The analysis was conducted at acceleration voltages of 3kV.

#### In vitro dissolution tests

##### Intrinsic dissolution test

The intrinsic dissolution rate (IDR) is stated as the dissolution rate of a pure active compound following compaction under the condition of constant surface area [28]. The equivalent of 50 mg of CXB in each sample was accurately weighed. The powder mixtures were compressed using a hydraulic press (Speca Press., UK) at 1 ton for 1 min. Pure CXB compacts were used for reference. After compression, the discs were assembled in the shafts of the dissolution apparatus. The dissolution medium used was 250 mL of pharmacopoeial phosphate buffer solution (PBS) at pH 6.8 with 30% (V/V) PEG 400, at a temperature of 37 ± 1 °C and the intrinsic dissolution test was conducted for 5 h at a rotation speed of 250 rpm. Sink conditions were maintained throughout the experiment. 1 mL of samples were collected at predefined time intervals (0.25, 0.5, 0.75, 1, 1.5, 2, 2.5, 3, 3.5, 4, and 5 h) from the dissolution vessels and replenished with the same volume of fresh medium. All samples were filtered through 0.22 µm pore-sized membrane filters and analyzed by HPLC (see Section “High-performance liquid chromatography determination of celecoxib”). To calculate the IDR, the cumulative amount of drug per unit area of the compact must be plotted against time. Linear regression is then performed, and the intrinsic dissolution rate is determined from the slope of the regression line, expressed in terms of the dissolved mass of a substance per time per exposed area [29, 30]. If the graph obtained presents curvature, only the initial linear region must be considered for the calculation of this parameter [31]. Enhancement ratios (ER) were also considered and calculated according to

3 $$\text{ER}\,=\,{\text{IDR}}_{\text{ASDs}}/{\text{IDR}}_{\text{CXB}}$$

##### Dissolution studies

In vitro dissolution testing simulates and predicts the in vivo performance of oral dosage forms in the gastrointestinal tract [32]. Tablets containing the test product were prepared by combining the ASD powder, lactose, and magnesium stearate and were obtained by direct compression in a hydraulic press (Speca Press., UK) at 5 tons, presenting a total weight of 342 mg (10 mg CXB). Tablets containing pure API, lactose, and magnesium stearate were prepared in the same conditions as a reference, weighing ~369 mg (equivalent to 10 mg of CXB). Each excipient was previously sieved (180 µm).

Drug release behavior of amorphous solid dispersions in tablets containing 10 mg of CXB was performed using USP Apparatus II (paddle apparatus) [33] with a rotation speed of 75 rpm. The dissolution medium used was 250 mL of pharmacopoeial phosphate buffer solution at pH 6.8 with 30% (V/V) PEG 400 at a temperature of 37 ± 1 °C to simulate the conditions of intestinal fluid without enzymes. The test was conducted for 8 h, and sink conditions were maintained throughout the whole experiment. 1 mL of samples were collected at predefined time intervals (0.25, 0.5, 0.75, 1, 1.5, 2, 3, 4, 6, and 8 h) from the dissolution vessels and replenished with the same volume of fresh medium. All samples were filtered through 0.22 µm pore-sized membrane filters and analyzed by HPLC (see “High-performance liquid chromatography determination of celecoxib” section). Dissolution profiles were achieved by plotting the percentage of drug release against time, calculated according to

4 $$\mathrm{Release\;(\%)\,=\,(Drug\,amount\,in\,}\mathrm{vessel)/(Total\,amount\,of\,drug\,in\,tablet) }\,\times\,\text{100}$$

#### Ex vivo permeability studies in Ussing chambers

##### Animals and tissue preparation

Adult RNU rats aged between 10–12 weeks and weighing 200–250 g were sourced from Charles River Laboratories (Lyon, France). They were housed in local animal facilities under controlled environmental conditions, including a 12-h light/dark cycle, a temperature maintained at 20 ± 2 °C, and a relative humidity of 50 ± 5%. All animal experiments were conducted in strict compliance with international regulations of the European Union (European Directive 2010/63/EU regarding the protection of laboratory animals used for scientific purposes) and were in accordance with the Portuguese law on animal welfare (Decreto-Lei 113/2013). The experimental protocols were thoroughly reviewed and approved by the Portuguese National Authority for Animal Health, Phytosanitation, and Food Safety (DGAV—Direção-Geral de Alimentação e Veterinária, Lisbon, Portugal), with the project reference "0421/000/000/2023". Effort was made to minimize both the number of animals utilized and any potential suffering they might endure.

Rat intestinal tissue was chosen as it has been widely utilized in intestinal drug permeability studies [34–36]. Additionally, mouse models have also been employed for similar purposes [37]. Fasted rats were sacrificed by decapitation, and the entire small intestine was rapidly removed and flushed with a PBS solution of pH 7.4. To minimize the tissue damage during the preparation, the intestine was allowed to cool for approximately 10 min. Afterward, jejunum segments with approximately 2 cm were cut along the mesenteric border and carefully placed in vertical Ussing chambers (Harvard Apparatus Inc., Holliston, MA, U.S.A.) with 0.64 cm^2^ of exposed area. Each side of the tissue was bathed with 1.5 mL of PBS solution with a pH of 7.4 and pH 6.8 for the acceptor (serosal membrane) and donor (mucosal membrane) compartments, respectively. Thereafter, chambers were screwed tightly and kept at 37 °C for the entirety of the experiment.

##### Ex vivo permeation experiment

Ex vivo permeation experiments were conducted following a 20 min equilibration period. 1.5 mL of drug solution composed of PBS pH 6.8 at a concentration of 10 mg/mL was inserted in the donor compartment. The serosal membrane was kept with PBS pH 7.4 with PEG 400, 70:30 (% V/V). Samples (200 µL) were collected from the acceptor compartment at 0.25, 0.5, 1, 2, and 3 h and analyzed by HPLC (see Section “High-performance liquid chromatography determination of celecoxib”). After each sample collection, 200 µL of fresh PBS 7.4 with PEG 400 medium, maintained at 37ºC, was replaced in the acceptor compartment.

To evaluate tissue integrity during the study, a stock solution (37.5 mg/mL) of fluorescein disodium salt in PBS pH 6.8 was previously prepared, and 20 µL was added in each donor compartment to obtain a final concentration of 0.05% (w/V) in the chamber. Samples of 100 µL were collected from the acceptor compartment to a microplate at the time points previously mentioned, and the same volume of fresh medium was replaced. The collected samples were analyzed by fluorometry (λ_excitation_: 485 nm; λ_emission_: 515 nm) in a Synergy HT Multi-Mode Microplate Reader (BioTeK, UK). Standard calibration curves were prepared for fluorescein to proceed to its quantification. The apparent permeability (P_app_) of CXB was calculated as

5 $${P}_{app}\;=\;\frac{Q}{C\;\times \;A\;\times \;t}$$

where Q (µmol) is the total amount of drug that permeated to the receiver compartment throughout the incubation time, C (µmol/mL) is the initial drug concentration in the donor compartment, A (cm^2^) is the diffusion area of the Ussing chamber, and t (s) is the duration of the experiment.

##### Membrane viability studies

To assess the viability of cells within the jejunum membrane following the permeability assay, the MTT (3-(4,5-dimethylthiazol-2-yl)-2,5-diphenyltetrazolium bromide) method was employed. After the permeability assay, rat intestinal membranes were incubated with 500 μL of MTT solution (5 mg/mL in PBS at pH 7.4) at 37 °C under stirring for 3h. As a control, rat intestinal membranes in PBS underwent a parallel incubation during the same period. Post-incubation, MTT was removed, and the tissue was rinsed in acidified isopropanol (1 μL concentrated hydrochloric acid per 1 mL isopropanol) to solubilize formazan crystals. Formazan absorbance was then measured at 570 nm and 620 nm using a Synergy HT Multi-Mode Microplate Reader (BioTeK, UK). Cell viability (%) was subsequently calculated and compared to the untreated control.

#### High-performance liquid chromatography determination of celecoxib

The quantification of CXB was performed using a previously validated HPLC method [20]. A Shimadzu LC-2010HT apparatus equipped with a quaternary pump (LC-20AD), an auto-sampler unit (SIL-20AHT), a CTO-10AS oven, and a SPD-M2OA detector was used. The analytical column used was Kinetex^®^ EVO C18, with a 5 mm particle size, 4.6 mm internal diameter, and 150 mm length, operated at an oven temperature of 35ºC. The mobile phase was composed of a mixture of 2% (V/V) glacial acetic acid:acetonitrile (50:50, % V/V) and was eluted at an isocratic flow rate of 1.2 mL/min. A run time of 10 min was established, and CXB was eluted at 7.5 min. The UV-Vis detection was carried out at 250 nm, and an injection volume of 10 μL was used for all samples. The results were further processed using the Shimadzu LC-solution version 1.12 software.

#### Statistical analysis

Statistical significance was evaluated using Student's t-test. A value of *p *< 0.05 was considered significant. This analysis was performed with GraphPad Prism PRISM 8.3.0 (GraphPad Software, San Diego, CA, USA).

## Results and discussion

### Definition of the quality target product profile

Following the principles of QbD, pharmaceutical development starts with the establishment of the quality target product profile (QTPP). This encompasses the desired attributes related to quality, effectiveness, and safety [38, 39]. Table 4 defines the QTPP based on quality prospects, scientific knowledge, and research interpretations.

**Table 4:** Quality target product profile and critical quality attributes (CQAs) for ASD tablets

**QTPP Elements**	**Target**	**Justification**
**Dosage form**	Tablet	Easy processability
**Dosage design**	Amorphous solid dispersion	To improve solubility and bioavailability
**Route of administration**	Oral dosage form	Common administration route of celecoxib Patient compliance
**Dosage strength**	10 mg	Easily scaled to other dosage strengths
**ASD powder**	Maximum % yield	To achieve higher drug loading in ASD
**Release**	Enhanced intrinsic dissolution rate	To improve dissolution, hence facilitating faster exposition to the dissolution medium and increased bioavailability
**Shelf-life**	≥ 3 years	In agreement with already marketed products
**Storage conditions**	Ensure drug product stability	Compliant with ICH Q1

### Risk estimation matrix

Risk assessment (REM) is defined as the identification of threats and the evaluation of risks associated with exposure to those threats [40]. The starting point of risk assessment is compiling the variables that could impact product CQAs and thus generate quality failure. The REM presented in Table 5 displays a qualitative risk estimation revealing the potential risk of process parameters.

**Table 5:**
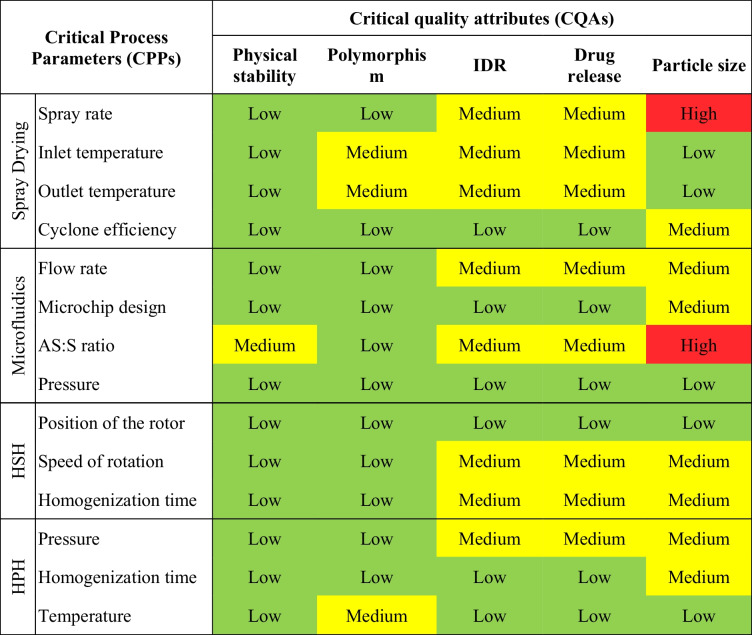
Initial REM presenting risk assessment levels of ASD formulation and the respective process parameters

*IDR* Intrinsic dissolution rate, *HSH* High-shear homogenization, *HPH* High-pressure homogenization, *Low* low-risk parameter, *Medium* medium -risk parameter, *High* high-risk parameter

Despite addressing formulation aspects, the AS:S ratio was herein considered as a CPP, given the control ensured by the technique upon the mixture of both phases

### Selecting the polymers

**Fig. 1:**
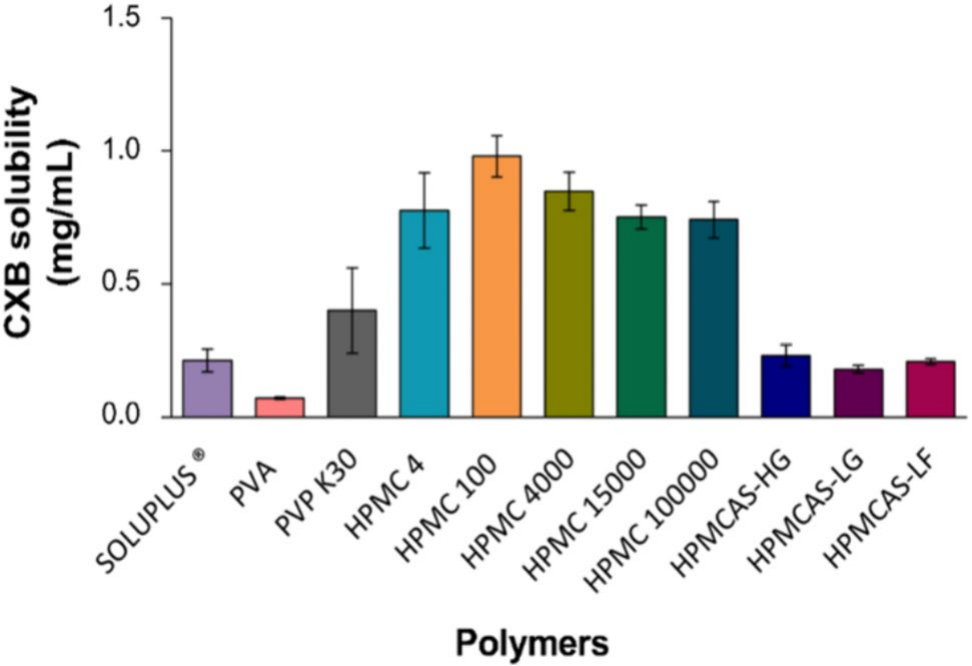
Concentration of CXB obtained from the ethanolic solutions of polymers. Key: PVP – polyvinylpyrrolidone; PVPVA - polyvinylpyrrolidone-co-vinyl acetate; HPMC - hydroxypropyl methylcellulose; HPMCAS - hydroxypropyl methylcellulose acetate succinate. The results are expressed in mean ± standard deviation (SD) (n = 3)

The selection of an optimal polymer is a critical aspect of amorphous solid dispersions formulation development. Polymers, as a matrix-forming excipient for these systems, can be involved in the recrystallization inhibition of amorphous active substances by exhibiting higher viscosity when it is below the glass transition temperature (T_g_) since a viscous polymeric matrix can help to maintain this kinetic stabilization, or through drug-polymer interactions. The T_g_ of an ASD system often falls between the T_g_ of the drug and the polymer. Because the T_g_ of the drug is usually lower than the T_g_ of the polymers used, ASDs benefit from the presence of a polymeric carrier in the formulation, since it helps increasing the T_g_ of the system [41–43]. A solvent evaporation technique was used, integrating different polymers and CXB to narrow down the initial selection to a few polymer-drug combinations. Solubilization capacity, measured by HPLC, was the pivotal parameter to evaluate since the solubility of the drug in the polymer has a direct impact on stabilizing the drug in its amorphous form, meaning that higher solubility is related to increased amorphicity [44]. The screening of polymers yielded the following results (Fig. 1).

Based on the experimental findings, it was observed that the five different HPMCs show the highest drug solubilization in comparison to the further polymers, emphasizing HPMC 100. Among the HPMCAS polymers, which differ from each other in their capacity to dissolve in different pH values, HPMCAS-HG emerged as the most favorable option. PVP K30 demonstrates the highest solubilizing capacity among non-cellulose derivative polymers. Stemming from the analysis of Fig. 1, HPMC 100 was selected as it demonstrated the highest capacity to solubilize CXB, and HPMCAS-HG was chosen due to its enteric coating ability, possibly granting a sustained release profile [45]. PVP K30, a commonly used hydrophilic polymer in ASD development, was also selected. Despite not displaying a pronounced solubilization of CXB, Soluplus^®^ was also taken into consideration as a promising polymer due to its amphiphilic crystallization inhibition capacity [46].

### Pre-formulation studies

Following the screening phase and identification of the most promising polymers, the polymers were tested individually and in combination. Binary polymeric combinations were prepared by pairing the selected polymers with each other. The aim of these combinations was to assess and comprehend their impact on particle size and polydispersity index. When formulating ASDs, the combination of two different polymers in a ternary system (drug + polymer 1 + polymer 2) can offer advantages in maintaining supersaturation and achieving an optimal dissolution rate [47]. The chosen manufacturing techniques for the pre-formulation studies were high-shear homogenization (HSH) to produce drug-polymer dispersions (0.25% w/V; 1:1:1) with an AS:S ratio of 50:50 (% V/V) and a combination of HSH followed by high-pressure homogenization (HPH), to assess the possibility of further reducing particle size. Table 6 displays the preliminary results of the developed dispersions. Particle size analysis was conducted using DLS.

**Table 6:** Particle size and respective polydispersity index of preliminary ASDs

**F**	**Polymer A**	**Polymer B**	**HSH**		**HPH**	
			**Particle Size (nm)**	**PdI**	**Particle Size (nm)**	**PdI**
**DP1**	Soluplus	-	206	0.351	322	0.382
**DP2**	PVP K30	-	> 1000	0.606	*	*
**DP3**	HPMC 100	-	> 1000	~1	> 1000	1
**DP4**	HPMCAS-HG	-	> 1000	0.924	*	*
**DP1P2**	Soluplus	PVP K30	101	0.210	180	0.217
**DP1P3**	Soluplus	HPMC 100	117	0.185	217	0.387
**DP1P4**	Soluplus	HPMCAS-HG	305	0.229	324	0.171
**DP2P3**	PVP K30	HPMC 100	> 1000	0.690	> 1000	0.962
**DP2P4**	PVP K30	HPMCAS-HG	191	0.153	159	0.206
**DP3P4**	HPMC 100	HPMCAS-HG	> 1000	~1	*	*

*HSH* High-shear homogenization, *HPH* High-pressure homogenization, *F* Formulation, *D* Drug (Celecoxib), *P* Polymer, *P1* Soluplus, *P2* PVP K30, *P3* HPMC 100, *P4* HPMCAS-HG

*Due to a visible phase separation and agglomeration of the formulation obtained from HSH, HPH was not performed

According to the experimental data obtained, the synergistic blending of two different polymers yields advantages in reducing the particle size and polydispersity index. However, it is noteworthy that high-pressure homogenization did not provide advantages in reducing particle size; instead, it leads to an increase in particle size in all the tested formulations, except for DP2P4. This outcome aligns with the known limitations of this technique, which the kinetics of particle coalescence can explain. Beyond a certain threshold, an increase in either the homogenization pressure or the time of processing can lead to an increase in particle size due to higher kinetic energy and the increased collision [48]. Similarly, the polydispersity index, a parameter that assesses the uniformity of particle sizes within a sample, increased in all formulations except for DP1P4. This indicates that HPH led to increased particle sizes and contributed to the development of more heterogeneous formulations. Consequently, HPH will not be performed in the upcoming formulation processing stages.

Furthermore, it was assessed that the polymeric combination obtained in DP2P4 exhibited the highest efficacy in inhibiting precipitation. This may be attributed to the contribution of HPMCAS-HG, which has been demonstrated to be a useful polymer in the stabilization of solid dispersions [45].

### Optimizing the composition

The selection of optimal conditions for the development of ASDs underwent a three-level experimental design with two variables. The most critical variables were identified, including the drug:polymers ratio and the total solid content. The choice of drug:polymers ratio is an important factor that influences the physical stability as well as the dissolution performance of these systems. Solid content is significant for product properties formulated via solvent evaporation since it can have an impact on the solution viscosity, consequently affecting the drying process and final product [49, 50].

According to the design, a total of 9 experiments were generated and performed, and their responses are represented in Table 7. The dispersions were prepared in the high-shear homogenizer, with an AS:S ratio of 50:50 (% V/V). Particle size analysis was conducted using DLS, while celecoxib assay was determined using HPLC. The polynomial coefficients, gathered in Table 8, were determined to assess the influence of each variable and their respective interaction, according to Eq. 1. It is fundamental to reflect that a higher coefficient magnitude indicates a stronger effect on the system, whereas a negative coefficient indicates that an increase in the parameter level leads to a reduction of the respective dependent variable [51].

**Table 7:** Three-level, two variable, 3^2^, full factorial design for optimizing ASDs formulation process

**F**	**Independent variables**		**Dependent variables**		
	**Total solid content % (w/V)**	**Drug:P1:P2 ratio**	**Particle Size (nm)**	**PdI**	**Assay (mg/mL)**
**1**	2.25	2:0.5:0.5	> 1000	0.771	25.4 ± 0.5
**2**	2.25	1:1:1	218 ± 7	0.191	13 ± 1
**3**	0.75	1:1:1	264 ± 11	0.164	5.8 ± 0.6
**4**	0.75	1.5:0.75:0.75	429 ± 147	0.476	7.8 ± 0.5
**5**	0.75	2:0.5:0.5	> 1000	0.950	10.1 ± 0.1
**6**	1.50	1:1:1	238 ± 36	0.118	10.8 ± 0.4
**7**	1.50	1.5:0.75:0.75	> 1000	0.817	14 ± 2
**8**	1.50	2:0.5:0.5	562 ± 89	0.734	20 ± 3
**9**	2.25	1.5:0.75:0.75	251 ± 49	0.556	21 ± 2

*F* Formulation, *PdI* Polydispersity index

The results are expressed in mean ± SD (n = 3)

**Table 8:** Parameters of the response surface for particle size, polydispersity index, and assay of ASDs

	**Particle Size**		**PdI**		**Assay**	
	**Estimate**	***p*-value**	**Estimate**	***p*-value**	**Estimate**	***p*-value**
**β**_**0**_	762.1	0.0677	0.642	< 0.0001	15.33	< 0.0001
**β**_**1**_	151.2	0.4929	-0.012	0.7623	5.999	< 0.0001
**β**_**2**_	875.8	0.0006	0.330	< 0.0001	4.426	< 0.0001
**β**_**12**_	293.9	0.2804	-0.051	0.2937	1.995	0.0004
**β**_**11**_	176.1	0.6437	-0.0385	0.5745	-1.338	0.0574
**β**_**22**_	236.1	0.5360	-0.1285	0.0708	-0.135	0.8403
**R**^**2**^** Adj**	0.3446		0.7347		0.9381	

*PdI* Polydispersity index, *β*_*0*_ Intercept, *β*_*1*_ Total solid content (%), *β*_*2*_ Drug:P1:P2 ratio, *β*_*12*_ Total solid content (%) * Drug:P1:P2 ratio, *β*_*11*_ Total solid content (%) * Total solid content (%), *β*_*22*_ Drug:P1:P2 ratio * Drug:P1:P2 ratio

Table 8 indicates that isolated coefficient terms are statistically significant in the vast majority. Scrutinizing the impact of the individual coefficient terms over particle size, it is evident that the drug:polymers ratio (β_2_) assumes major relevance, followed by the total solid content (β_1_). This implies that the drug:polymer ratio influences particle size more than any other studied independent variable. In what concerns the interaction term (β_12_), it reveals a bigger impact on particle size than β_1_ indicating a synergistic effect. This demonstrates that for higher total solid content and drug:polymers ratio, the interaction favors particle size. For PdI and drug assay, the coefficient terms did not exhibit a great impact. However, the total solid content influences the assay positively.

**Fig. 2:**
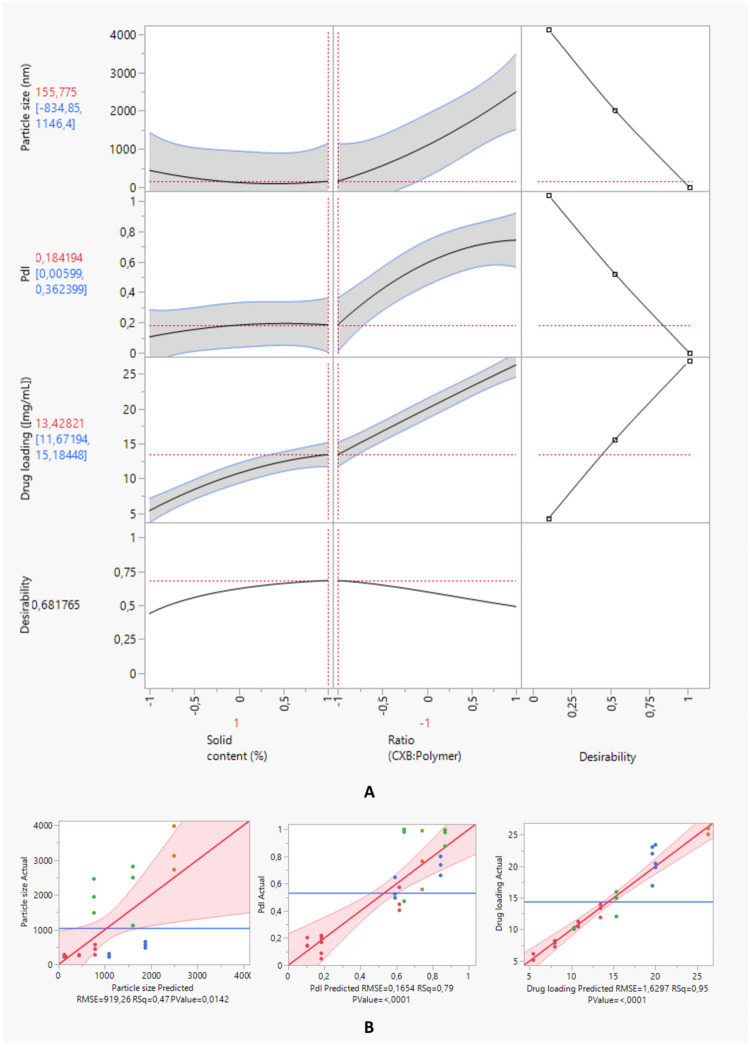
Actual by predicted plots for the responses displaying a better goodness of fit. **A1** Actual by the predicted plot for particle size; **A2** Actual by the predicted plot for polydispersity index; **A3** Actual by the predicted plot for drug assay; **B** Overall desirability for ASDs process parameters optimization, according to the target (increase or decrease) imposed per CQA. The last row of plots displays the desirability trace for each factor. The overall desirability for all responses is defined as the geometric mean of the desirability functions of the individual responses

Analyzing Fig. 2A, it should be noted that for a perfect fit, all the points should be on the Y = X diagonal line. The horizontal line represents the mean of each response. A 95% confidence region is shown on leverage plots, where the curves visually indicate whether the test is significant at the α = 0.05 level. The model test is not considered significant if the red region contains the horizontal line. If the curves cross the line, the effect is significant. Generally, the dependent variables were well adjusted, presenting a *p*-value < 0.05.

The desirability approach was employed to target all CQAs, namely minimizing particle size, minimizing polydispersity index, and maximizing drug assay. According to the desirability (D) function, the measured quality characteristics of each predicted response are converted into a dimensionless desirability value, comprised between 0 and 1. A value of D different from 0 reveals that all responses are within a desirable range. As this value gets closer to 1, the combination of different criteria is considered optimal. *Per se*, when the response values are close to the target ones, a D = 1 is obtained [20, 52]. Figure 2B shows that the maximum desirability (0.682) was found for the formulation containing the highest total solid content (level 1, 2.25% w/V) and the lowest drug-to-polymers ratio (level -1, 1:1:1), corresponding to F2.

### Inspecting the stability

**Fig. 3:**
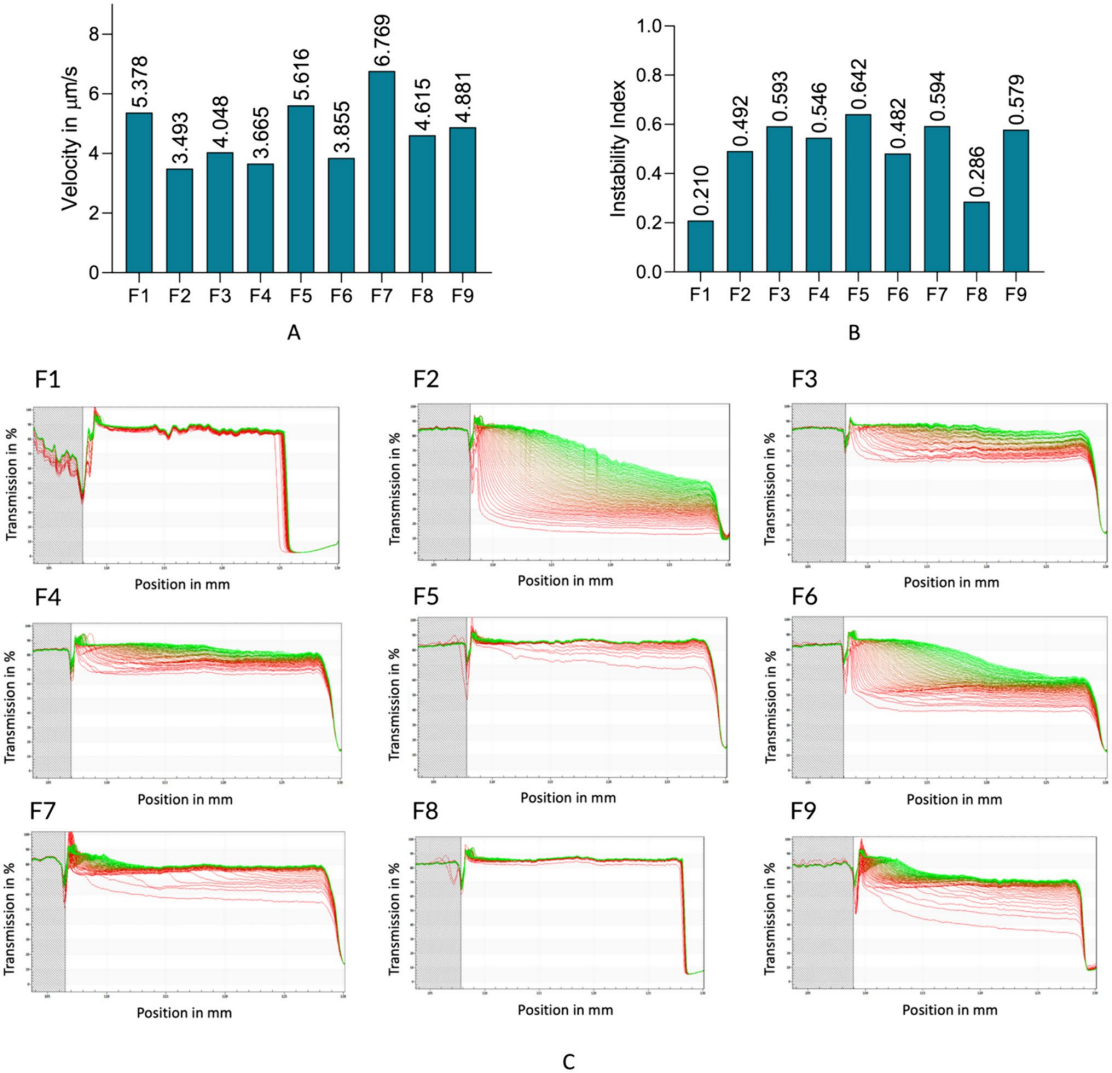
Analytical centrifugation results provided by LUMiSizer. **A** Instability indexes of F1-F9; **B** Sedimentation velocity of F1-F9; **C** Transmission profiles (transmission % *vs.* position in mm) provided by the SEPView software. Red indicates the first profile, while green indicates the last profile

A predictive assessment of the physical stability of the formulations F1-F9 was performed on the LUMiSizer, which applies centrifugal force to accelerate potential destabilization phenomena, such as sedimentation [48, 53]. Instability index and sedimentation velocity are two of the results retrieved by this equipment, depicted in Fig. 3. The instability index, a quantitative parameter that ranges from 0 (more stable) to 1 (more unstable), is analyzed in Fig. 3A. It is perceivable that formulations F1 and F8 stand out as the ones demonstrating greater stability. However, these values may be influenced by the rapid precipitation of these formulations prior to the analysis, leading to lower instability indexes. As shown in Fig. 3C, the transmission profiles of these two formulations (F1 and F8) remained very constant during the analysis. Regarding sedimentation velocity, portrayed in Fig. 3B, F2 indicates the lowest value. However, in Fig. 3C, F2 stands out as the most polydisperse sedimentation profile, implying that sedimentation occurred slowly and at a controlled rate. Gathering the results from analytical centrifugation and experimental design, it is evident that F2 exhibits the most promising developmental conditions.

### Tailoring polymer composition based on the intrinsic dissolution rate of CXB-ASDs

The impact of different polymers and drug-polymer interactions plays a role in the dissolution rate of ASDs, which depends on the polymer release rate and the strength of the interactions [54]. Accordingly, intrinsic dissolution was studied to attain a comprehensive understanding of polymer selection and drug/polymer interactions of these systems on the drug release of ASDs. PVP K30, HPMC 100, HPMCAS-HG, and Soluplus^®^ were considered polymers. Soluplus^®^ is particularly notable for its ability to enhance the solubility of poorly soluble drugs due to its amphiphilic nature. It has exhibited great potential in improving the solubility, dissolution, and bioavailability of drugs with various physicochemical properties, as well as enhancing drug wettability. For this reason, Soluplus^®^ was incorporated in all polymeric combinations [7]. ASDs were prepared using microfluidics-on-a-chip equipment with an AS:S ratio of 50:50 (% V/V), following the formulation compositions displayed in Table 2. After conducting a series of experiments to optimize the flow rate, it was determined that the optimal flow rate for both streams (antisolvent and solvent) is set at 500 µL/min. After obtaining the CXB-ASD formulations, the solvent was removed in a static bed stove under 40 °C for 48 h, thus obtaining the powder used in the IDR studies. The obtained IDR and their respective enhancement ratios (ER) are depicted in Table 9.

**Table 9:** Overview of the intrinsic dissolution rates of binary polymer combination ASDs obtained using the microfluidics-on-a-chip technique (n = 3 was conducted per experiment)

	**IDR (µg min**^**-1**^** cm**^**-2**^**)**	**ER**
**CXB**	4.49	1
**ASD-P1P2**	3.44	0.77
**ASD-P1P3**	5.32	1.18
**ASD-P1P4**	3.93	0.88
**ASD-SD**	4.85	1.08
**ASD-MF**	118.33*	26.35*

*ASD* Amorphous solid dispersion, *P* Polymer, *P1* Soluplus, *P2* PVP K30, *P3* HPMC100, *P4* HPMCAS-HG, *SD* Spray Drying, *MF* Microfluidics-on-a-chip

*p-value < 0.05

Based on the data represented in Table 9, the enhancement ratios achieved with the developed ASDs were not found to be satisfactory. Previous studies have reported that when an ASD comes in contact with water, phase transformations can occur, a phenomenon known as water-induced amorphous-amorphous phase separation (AAPS) [55–57]. The hydrated ASD matrix separates into an insoluble amorphous drug-rich phase and a soluble polymer-rich phase. Several studies have suggested that AAPS can lead to drug crystallization, as the polymer capacity of inhibiting crystallization has been lost due to separation, resulting in hindered drug release [58, 59]. Additionally, the compacts obtained from the intrinsic dissolution disks exhibited swelling after the conclusion of the study. It could also be hypothesized that the swelling phenomena could entrap the API and hamper its release, which might also explain the low IDR observed, as the outermost layer did not facilitate thorough drug release [60]. Moreover, PVP K30 (< 0.5 mg/mL) and HPMCAS-HG (< 0.5 mg/mL) provide a lower drug solubility, yielding drug precipitation and limiting the intrinsic dissolution of CXB-ASD compared to CXB. However, in the presence of HPMC 100, where the solubility of CXB is ~1 mg/mL, the CXB-ASD exhibited a higher intrinsic dissolution rate.

Considering this, in an attempt to further increase the CQAs referred to in the QTPP, an investigation was conducted to explore the approach of combining three of the abovementioned polymers to address the challenge of low dissolution rates. Two separate approaches to manufacturing ASDs were concurrently explored: microfluidics-on-a-chip and spray drying. Microfluidics-on-a-chip, chosen for its innovativeness, employs high-shear forces and continuous fluid processing through a microchip to generate small particles and achieve a homogeneous dispersion. In contrast, spray drying, a conventional method for ASD production, was employed as a comparative technique. This method involves atomizing a drug-polymer solution and drying the ASDs in a single step. Each technique presents unique technological advantages, justifying a comprehensive investigation of their efficacy in enhancing dissolution rates and improving drug delivery. Thus, CXB, HPMC 100, HPMCAS-HG, and Soluplus^®^ were combined each at a concentration of 0.75% (w/V) and a ratio of 1:1:1:1. The intrinsic dissolution data for ternary polymer combination ASDs obtained using flow-focused microfluidization demonstrates the fastest IDR for all the studied polymeric combinations and manufacturing techniques, resulting in a 26-fold increase when compared to pure CXB. Therefore, it can be concluded that implementing the microfluidics-on-a-chip technique in developing CXB-ASDs with ternary polymeric matrices yielded the most favorable outcomes compared to all other tested combinations and manufacturing techniques.

Table 10 displays the particle sizes of CXB, ASD-SD, and ASD-MF formulations. The liquid dispersions that originated ASD-SD and ASD-MF presented a mean particle size of 193.5 nm (PdI = 0.555) and 160.5 nm (PdI = 0.389), respectively, revealing nanometric properties and a degree of heterogeneity, confirmed by SEM analysis (see Fig. 5). The ASD-MF formulation presents overall greater particle sizes after drying, indicating that static bed drying may not be the most suitable downstream drying process for this preparation. On the other hand, spray drying is known for the opportunity of particle engineering and yields fine particles (ASD-SD), which is in harmony with the data presented in Table 10 [61].

**Table 10:** Particle size measurements of pure celecoxib and amorphous solid dispersions obtained by laser diffraction

**Sample**	**Dv(10) µm**	**Dv(50) µm**	**Dv(90) µm**	**Span**	**D [3;2] µm**	**D [4;3] µm**	**Residual %**	**Weighted Residual %**
**CXB**	3.88	14.7	39.0	2.39	7.13	18.1	0.10	0.10
**ASD-SD**	1.82	9.24	24.4	2.44	3.53	11.6	0.09	0.08
**ASD-MF**	9.68	29.6	46.3	1.24	20.5	28.7	1.00	0.97

*CXB* Celecoxib, *ASD-SD* Amorphous solid dispersion obtained by spray drying, *ASD-MF* Amorphous solid dispersion obtained by microfluidics-on-a-chip

Results are expressed in mean (n = 6)

A comparative analysis of the use of microfluidics-on-a-chip for obtaining CXB-ASDs points out several technological aspects of previous studies [12, 62–64]. Considering the amount of CXB, the studies already published report a lower amount of CXB in the ASD formulations. The ASDs developed in the present study contain 33% of drug loading, compared to 20%, 10%, or less than 10% reported in the literature [12, 62, 63]. Another distinguishing factor is the production method of ASD formulations. While some studies utilize hot melt extrusion or spray drying, both methods have inherent disadvantages that microfluidics can address. For instance, although hot melt extrusion (HME) is a well-established manufacturing technique for ASDs, characterized by the melting and mixing of drug and polymer components under high temperature and shear forces, it is limited by potential thermal degradation of heat-sensitive drugs and polymers, as well as challenges in achieving high drug loading levels, and control over particle size and morphology. Similarly, spray drying, despite its widespread use, may yield poor encapsulation efficiency, agglomeration, and variability in particle size distribution. For these reasons, the use of microfluidic systems could offer significant advantages. Microfluidics provides precise control over particle properties and enables continuous manufacturing, representing a substantial advancement for the industry. This innovation leads to enhanced process efficiency and reduced production costs.

### Thermodynamic and structural characterization of ASDs

**Fig. 4:**
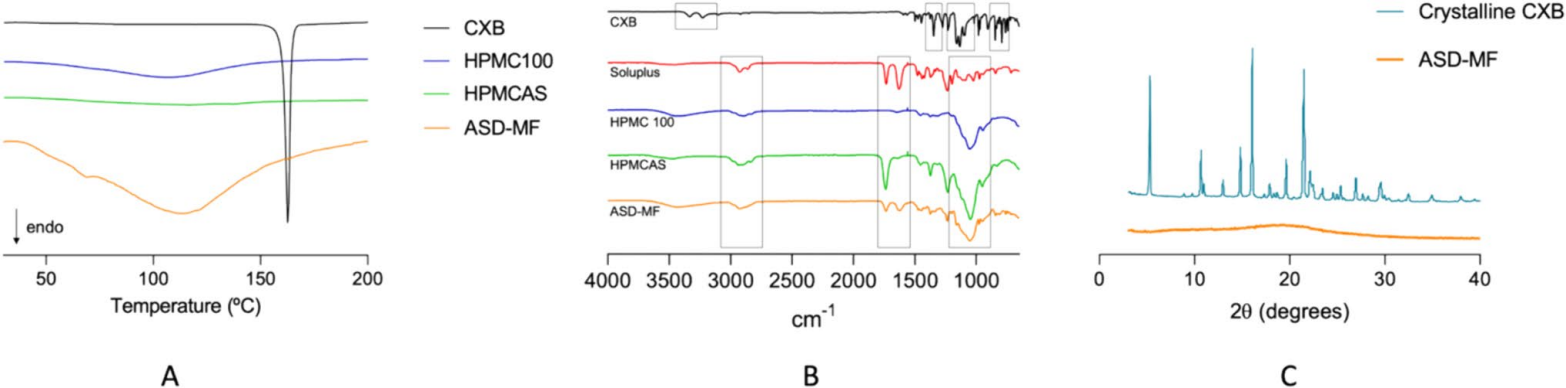
**A** Thermograms, **B** FTIR spectra, and **C** XRPD patterns of pure celecoxib and celecoxib amorphous solid dispersion. KEY: CXB = Crystalline celecoxib; HPMC = hydroxypropyl methylcellulose; HMPCAS = hydroxypropyl methylcellulose-acetate succinate; ASD-MF = Amorphous solid dispersion obtained by microfluidics-on-a-chip

Table 3 displays the melting temperature and glass transition temperatures of pure compounds. DSC experiments reveal that the endothermic peak associated with the melting of CXB was not present on the evaluated solid dispersion, as displayed in Fig. 4A.

A sharp melting endothermic peak of pure celecoxib is observed at 161 °C. A melting endotherm depression complemented by shifting to a lower temperature implies a significant degree of mixing, revealing that the drug is molecularly dispersed in the polymeric matrix. This data leads to the conclusion that this formulation presents CXB in a fully amorphous state and that CXB is completely miscible with the remaining excipients.

**Fig. 5:**
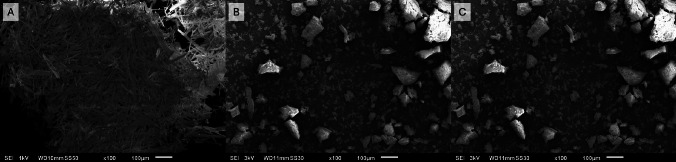
SEM images of crystalline celecoxib **A** and celecoxib amorphous solid dispersions. **B** and **C** correspond to 100 and 300-fold digital magnification of a representative area of the sample

The ATR-FTIR spectra obtained for pure compounds and ASD-MF formulation are represented in Fig. 4B. Analysis of these spectra enables comprehension of the molecular arrangements within the ASD-MF formulation and provides insight into the interactions between its components. The infrared spectroscopy (IR) spectrum of CXB displays absorption bands at 3223.36 and 3329.90 cm^-1^ assigned to the drug N−H stretching vibrations. The peaks at 1131.40 and 1345.60 cm^-1^ belong to the S=O stretching vibrations. N−H bendings are represented at 1562.71 cm^-1^, and the aromatic C−H bendings peak at 791.54 cm^-1^ [65]. Regarding the ASD-MF formulation, a broad band present at 3300 cm^-1^ is attributed to the presence of water. A peak at 2909 cm^-1^ is consistently observable in all polymers spectra and the ASD-MF formulation, representing C–H stretching vibrations. The peak at 1732 cm^-1^ is attributed to the C=O stretching vibrations. Both Soluplus^®^ and the tested formulation present a second peak at 1626 cm^−1^ due to a second carbonyl (C=O) group. The dominant peak in ASD-MF at wavelength 1050 cm^-1^ is due to the HPMC and HPMCAS contribution and is related to the C–O stretching vibration. The FTIR spectra provides a deep understanding of the structural characteristics and interactions within the system. The ASD formulation exhibited peaks resembling those of the polymers, suggesting strong interactions and miscibility of the drug within the polymeric matrix, as confirmed by the DSC analysis. Wide angle X-ray scattering patterns were obtained for pure CXB and the ASD-MF, as displayed in Fig. 4C. CXB is a highly crystalline powder that presents distinctive sharp diffraction peaks at 2 values of 5 º, 10.8°, 16°, and 21.5° that indicate its crystalline state. In contrast, ASD-MF does not display any diffraction peaks from crystalline CXB, demonstrating that all CXB in this formulation is in an amorphous state. These results are consistent with SEM analysis, in which CXB is presented in a fully amorphous state. The SEM images of ASD-MF are represented in Fig. 5. The lack of a well-defined crystal structure with a random arrangement of particles is observable alongside polyhedric structures. From a global appreciation, the SEM images agree with the amorphization evidenced from the XRPD and DSC analysis, when compared to pure CXB.

### Evaluating the dissolution behavior

**Fig. 6:**
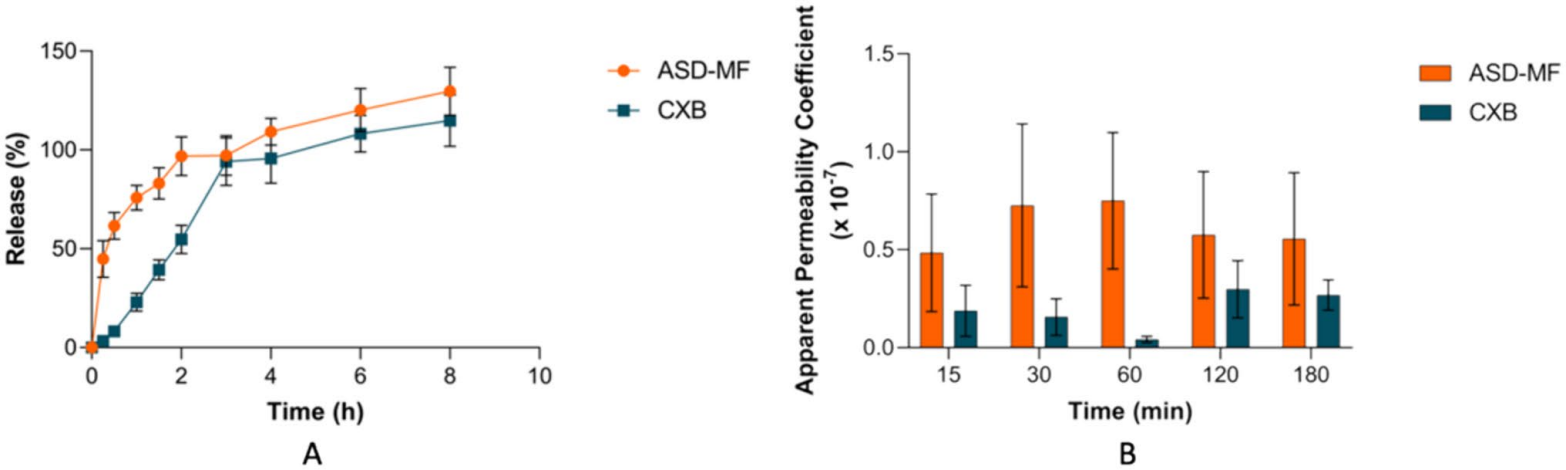
**A** Release profiles of celecoxib and celecoxib amorphous solid dispersions. **B** Apparent permeability of ASD-MF and CXB formulations. Key: CXB = celecoxib; ASD-MF = celecoxib amorphous solid dispersion with ternary polymeric matrix, prepared by means of microfluidics-on-a-chip. The results are expressed in mean ± SEM (6 ≤ n ≤ 8)

The dissolution behavior of the API and polymers from ASDs is critical for oral drug absorption. Even though the intricate mechanisms of dissolution of ASDs are not well understood, they have received increasing interest. When water enters the ASD matrix during dissolution, it could change the properties of the ASD, showing the importance of thoughtful formulation design to reach a balance between physical stability and drug dissolution. It has been hypothesized that the polymer-drug molecular interactions should be strong enough to shield the amorphous solid dispersions from water-induced phase separation and inhibit crystallization. Maintaining drug supersaturation, induced by ASD dissolution, is also significant for the enhancement of bioavailability [66, 67]. This work used the USP apparatus type II to dissolve ASD tablets. The release profiles of the developed formulation and the crystalline CXB are portrayed in Fig. 6A. The release percentage exceeding 100% in the ASD-MF formulation is attributed to a disparity in the dosing of the tablet content. Overall, the ADS-MF formulation exhibited a higher release rate than CXB used as a reference, a behavior consistent with the intrinsic dissolution rate. The improved dissolution rate can be attributed to the absence of molecular order and the increased energy associated with the amorphous form. The lack of a well-defined crystal lattice in the amorphous state enables a faster dissolution process since no energy is needed to overcome the crystalline structure. In contrast, crystalline materials require the disruption of the lattice structure for the active component to dissolve, leading to a comparatively slower dissolution rate [68, 69]. These results support the hypothesis that ASDs may increase the bioavailability of the drug when compared to the crystalline state.

Various mathematical models were employed to rationalize the drug release mechanisms using the DDSolver software, and their parameters are shown in Table 11 [70]. The selection of a suitable model for dissolution data is critical, and the goodness of fit was assessed according to both the adjusted R-squared (R^2^ adj.) and the model selection criterion (MSC). A well-adjusted model should have an R^2^ adj. value closest to 1, and the highest MSC value determines the most appropriate model. Analyzing the data, the Weibull, Gompert and Korsmeyer-Peppas models provided the best fit for both the ASD-MF and the CXB tablets. A zero-order profile was not possible to be traced for this formulation. Focusing on the Korsmeyer-Peppas model, frequently used for determining the release kinetics of polymeric dosage forms, the value of the parameter c2 holds particular relevance in characterizing the drug transport mechanism [71]. Accordingly, c2 values close to 0.5 indicate a Fickian diffusion process, whereas values ranging from 0.5 to 1.0 suggest an anomalous (non-Fickian) transport. When the c2 value equals 1.0, it implies applying a zero-order model. Values larger than 1 point out to a Super Case-II transport [72]. It is worth noting that the estimation of this exponent only considers the initial 60% of the release. Applying this classification to the obtained results, it becomes evident that the ASD-MF formulation resembles a Fickian diffusion, specifically a hindered Fickian diffusion, characteristic of systems exhibiting a diffusive regime with impeded release [73]. The control formulation containing CXB reveals an exponent superior to 1.0, whose diffusion mechanism may be attributed to the hydrophilic groups in the polymeric chains. Erosion and polymer network relaxation are reported to regulate this mechanism, caused by solvent penetration which is a consequence of the formations of pores in the pharmaceutical form [74].

**Table 11:** Drug release kinetics parameters

**Params**	**Zero-order** ${\varvec{c}}1{\varvec{t}}$	**First-order** ${{\varvec{F}}}_{{\varvec{m}}{\varvec{a}}{\varvec{x}}}(1-{{\varvec{e}}}^{\left(-{\varvec{c}}1{\varvec{t}}\right)})$	**Higuchi** ${\varvec{c}}1{{\varvec{t}}}^{0.5}$	**Korsmeyer-Peppas** ${\varvec{c}}1{{\varvec{t}}}^{{\varvec{c}}2}$	**Weibull** ${{\varvec{F}}}_{{\varvec{m}}{\varvec{a}}{\varvec{x}}}(1-{{\varvec{e}}}^{\left(-\frac{{\left({\varvec{t}}\right)}^{{\varvec{c}}2}}{{\varvec{c}}1}\right)})$	**Gompertz** ${{\varvec{F}}}_{{\varvec{m}}{\varvec{a}}{\varvec{x}}}{{\varvec{e}}}^{{(-{\varvec{c}}1\times {\varvec{e}}}^{-{\varvec{c}}2\boldsymbol{*}\mathbf{log}({\varvec{t}})})}$
**ASD-MF**						
**c1**	-	1.184	54.40	74.04	1.838	1.747
**c2**	-	-	-	0.275	0.409	0.404
**F**_**max**_	-	113.3	-	-	178.0	431.1
**R**^**2**^	-	0.924	0.797	0.993	0.995	0.996
**R**^**2**^** adjusted**	-	0.914	0.797	0.992	0.994	0.994
**MSE_root**	-	11.32	17.43	3.550	3.074	2.919
**WSS**	-	1025	2735	100.8	66.13	59.66
**MSC**	-	1.489	0.7077	3.808	4.030	4.133
**CXB**						
**c1**	18.319	0.282	41.893	22.61	4.480	1.896
**c2**	-	133.9	-	1.291	1.726	2.982
**F**_**max**_	-	-	-	-	111.3	132.1
**R**^**2**^	0.812	0.966	0.896	0.999	0.993	0.987
**R**^**2**^** adjusted**	0.812	0.962	0.896	0.998	0.991	0.984
**MSE_root**	19.79	8.949	14.69	0.877	4.316	5.797
**WSS**	3524	640.7	1942	3.074	130.4	235.2
**MSC**	1.301	2.806	1.897	5.775	4.198	3.608

*c1* release rate constant, *c2* release exponent, *MSE_root* standard deviation of residuals, *WSS* weighted sum of squares, *MSC* model selection criterion

### Analyzing the ex vivo permeability

The prediction of drug intestinal absorption can be obtained through ex vivo permeability studies. The Ussing chamber system allows for a localized assessment of intestinal permeability [75]. Both the amorphous solid dispersion obtained by microfluidics-on-a-chip (ASD-MF) and pure CXB were tested, and the obtained results are represented in Fig. 6B. The ASD-MF reveals a higher value of permeability coefficient (P_app_) when compared to the drug in its crystalline state. Previous studies have revealed that amorphous solid dispersions may increase not only the drug solubility and dissolution rate but also the drug’s flux through the intestinal membrane [76, 77]. The observed permeability values for the amorphous solid dispersions demonstrated a 2-fold enhancement compared to pure CXB (Table 12). Moreover, membrane integrity was evaluated by monitoring the apparent permeability coefficient for sodium fluorescein, a paracellular marker. The values were below 0.13 × 10^-6^ cm/s, reflecting the full integrity of the membrane [78]. The intestinal fragments used were submitted to a cytotoxicity assay at the end of the permeability study performed in the Ussing chambers. Cell viability was assessed using the MTT technique compared to intestinal membranes that were not submitted to formulation. The MTT values demonstrated almost 100% cell viability, proving that the ASD formulation has a favorable safety profile and is not thought to be cytotoxic.

**Table 12:** Apparent permeability coefficient of ASD-MF, CXB, and respective enhancement ratios at 180 min

**Formulation**	**P**_**app**_** (cm.s) (×10**^**–7**^**)**	**ER**
**ASD-MF**	0.56 ± 0.28	2.06
**CXB**	0.27 ± 0.07	1

The results are expressed as mean ± SEM (5 ≤ n ≤ 6)

### Towards optimal conditions

Based on previous knowledge, a preliminary risk assessment was conducted to identify and predict high-risk variables that could influence the critical quality attributes (CQAs) of the ASD formulation. The risk assessment should be updated at different developmental stages. An updated REM is presented in Table 13. Following the results of the experiments, a notable shift in the risk assessment matrix was observed, particularly concerning spray drying and microfluidic processes according to the CQAs evaluated. Prior to experimentation, concerns regarding potential alterations to CQAs, including polymorphism, IDR (%), and drug release, due to variations in outlet temperature during the spray-dried process were prominent. However, the post-experimental analysis revealed that the outlet temperature remained consistent and did not exert any discernible impact on the identified CQAs (see Table 5 vs. Table 13). This observation displayed a significant reassessment of the associated risks, indicating a lower probability of variations on product quality originating from outlet temperature.

**Table 13:**
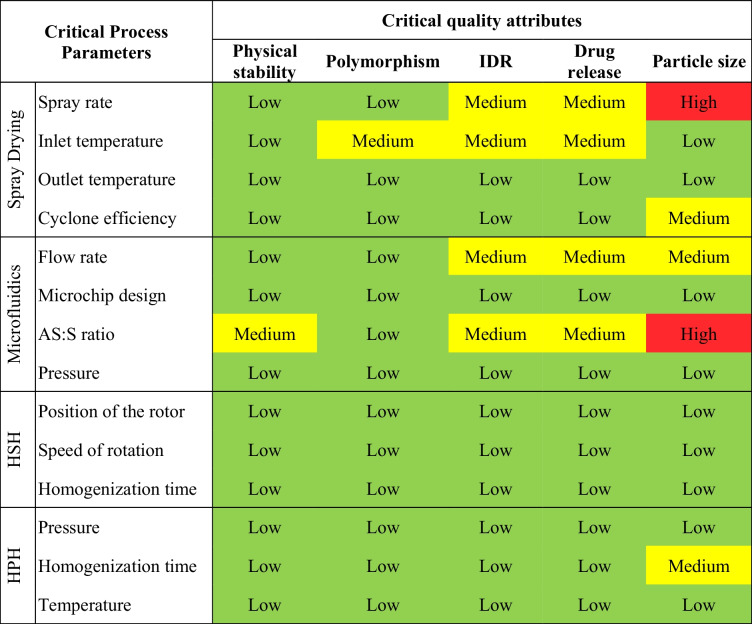
Updated REM presenting risk assessment levels of ASD formulation and the respective process parameters

*IDR* Intrinsic dissolution rate, *HSH* High-shear homogenization, *HPH* High-pressure homogenization, *Low* low-risk parameter, *Medium* medium risk parameter, *High* high-risk parameter

Despite addressing formulation aspects, the AS:S ratio was herein considered as a CPP, given the controlled ensured by the technique upon the mixture of both phases

## Conclusion

In the present work, amorphous solid dispersions were developed, resorting to different manufacturing techniques, such as antisolvent precipitation, high-shear homogenization, high-pressure homogenization, microfluidics-on-a-chip, and spray drying. Different polymeric combinations were assessed to understand their influence in the system, and different techniques were compared to obtain ASDs. Through quality by design optimization, a ternary polymeric combination was identified as highly effective in enhancing the intrinsic dissolution rate of the drug, resulting in a remarkable 22-fold increase compared to the binary polymer combination. Furthermore, the ASD formulation exhibited improved drug release rates, indicating its potential for overcoming bioavailability challenges associated with several APIs already approved in the market. The results obtained from dissolution studies were further substantiated by ex vivo analysis, emphasizing the robustness of the ASD-MF performance. The significant improvements observed in dissolution behavior (26-fold higher) were supported in the ex vivo studies, which doubled drug permeability compared to pure CXB. This consistency between dissolution and permeability outcomes underscores the possibilities of ASD-MF in improving drug release and absorption properties. Hence, this work addressed the bioavailability challenges that arise with various active substances, exploring a pathway to more efficient drug delivery systems.

Overall, this work is a supplement to the research that is being developed regarding the poor water solubility of new chemical entities, exploring the versatility of different methods and pathways to develop more efficient drug delivery systems. Bio-enabling technologies were implemented, hopefully serving as a guiding light for future pharmaceutical development, instigating new discussions and positive outcomes.

## Data Availability

The raw data required to replicate these findings are available on reasonable request from the corresponding author (C.V.). The data will be made available on request.
